# The Effects of Calcium-*β*-Hydroxy-*β*-Methylbutyrate on Aging-Associated Apoptotic Signaling and Muscle Mass and Function in Unloaded but Nonatrophied Extensor Digitorum Longus Muscles of Aged Rats

**DOI:** 10.1155/2020/3938672

**Published:** 2020-07-23

**Authors:** Brian T. Bennett, Junaith S. Mohamed, Stephen E. Alway

**Affiliations:** ^1^Laboratory of Muscle Biology and Sarcopenia, Department of Exercise Physiology, West Virginia University School of Medicine, Morgantown, WV, USA 26506; ^2^Center for Muscle, Metabolism and Neuropathology, Division of Rehabilitation Sciences, College of Health Professions, University of Tennessee Health Science Center, Memphis, TN, USA 38163; ^3^Laboratory of Nerve and Muscle, Department of Diagnostic and Health Sciences, College of Health Professions, University of Tennessee Health Science Center, Memphis, TN, USA 38163; ^4^Laboratory of Muscle Biology and Sarcopenia, Department of Physical Therapy, College of Health Professions, University of Tennessee Health Science Center, Memphis, TN, USA 38163; ^5^Department of Physiology, College of Medicine, University of Tennessee Health Science Center, Memphis, TN, USA 38163

## Abstract

Beta-hydroxy-beta-methylbutyrate (HMB), a naturally occurring leucine metabolite, has been shown to attenuate plantar flexor muscle loss and increase myogenic stem cell activation during reloading after a period of significant muscle wasting by disuse in old rodents. However, it was less clear if HMB would alter dorsiflexor muscle response to unloading or reloading when there was no significant atrophy that was induced by unloading. In this study, we tested if calcium HMB (Ca-HMB) would improve muscle function and alter apoptotic signaling in the extensor digitorum longus (EDL) of aged animals that were unloaded but did not undergo atrophy. The EDL muscle was unloaded for 14 days by hindlimb suspension (HS) in aged (34-36 mo.) male Fisher 344 × Brown Norway rats. The rats were removed from HS and allowed normal cage ambulation for 14 days of reloading (R). Throughout the study, the rats were gavaged daily with 170 mg of Ca-HMB or water 7 days prior to HS, then throughout 14 days of HS and 14 days of recovery after removing HS. The animals' body weights were significantly reduced by ~18% after 14 days of HS and continued to decline by ~22% during R as compared to control conditions; however, despite unloading, EDL did not atrophy by HS, nor did it increase in mass after R. No changes were observed in EDL twitch contraction time, force production, fatigue resistance, fiber cross-sectional area, or markers of nuclear apoptosis (myonuclei + satellite cells) after HS or R. While HS and R increased the proapoptotic Bax protein abundance, BCL-2 abundance was also increased as was the frequency of TUNEL-positive myonuclei and satellite cells, yet muscle mass and fiber cross-sectional area did not change and Ca-HMB treatment had no effect reducing apoptotic signaling. These data indicate that (i) increased apoptotic signaling preceded muscle atrophy or occurred without significant EDL atrophy and (ii) that Ca-HMB treatment did not improve EDL signaling, muscle mass, or muscle function in aged rats, when HS and R did not impact mass or function.

## 1. Introduction

Prolonged immobilization or disuse causes a rapid loss of muscle mass and force in aging populations. This is particularly problematic in the elderly, where this loss of muscle mass is already high (i.e., sarcopenia) [1–3] and further loss of muscle can lead to a decline in strength and may increase the risk of falls [4, 5]. Falls are clinically relevant to the elderly population as they are a leading cause of morbidity and mortality in older subject groups [6]. Furthermore, diminished muscle mass and strength (i.e., sarcopenia) in itself is associated with an increased risk of mortality and cognitive decline [7, 8]. For these reasons, it is important to develop novel treatments to reduce muscular atrophy in the elderly.

Muscle loss with disuse or bedrest in aging is due in part to the following: decreases in protein synthesis and increases in proteolysis in various limb skeletal muscles [9–20] including increases in collagen synthesis; and downregulation of ribosomes, oxidative metabolism, and mitochondrial gene transcripts in the vastus lateralis muscles of human [21]. Muscle loss is also associated with an increase in apoptotic signaling in myonuclei and satellite cells in fast- and slow-contracting limb muscles from older animals and humans [15, 22–27]. This reduces the number of myonuclei and/or myogenic stem cells (satellite cells) and thereby reduces the potential for muscle growth or repair [19, 28, 29]. Hindlimb suspension (HS) has been used widely as a preclinical model of atrophy to study a variety of skeletal muscle adaptations including reduced gravity, disuse, and reloading (R) following disuse [30–35]. HS has been shown to rapidly decrease muscle mass in plantar flexor muscles of rodents and this appears to have a mitochondrial role in muscle loss [36].

We have studied unloading in plantar flexor muscles of aged rats or mice for many years [29, 37–50]. This has included two studies [43, 51] that found a beneficial effect of beta-hydroxy-beta-methylbutyrate (HMB) for reducing wasting in the fast-contracting plantaris and slow-contracting soleus plantar flexor muscles with unloading. HMB is a naturally occurring metabolite of the essential branched-chain amino acid leucine with no known genotoxic effects [52]. Furthermore, HMB appears to be particularly beneficial for improving upper and lower body muscle mass and strength in humans that exercise [53–55], although it is less clear if HMB has an anabolic effect without an exercise or loading stimulus [53]. In addition, HMB has been shown to decrease proteolysis in muscles of persons with cancer cachexia and attenuate decreased protein synthesis in murine myotubes in culture in response to apoptotic stimuli [56, 57]. HMB appears to increase protein synthesis via the mTOR pathway [58, 59]. Our previous studies have found beneficial effects of the calcium form of HMB (Ca-HMB) to reverse HS-induced muscle atrophy that was superimposed on aging-associated sarcopenia [43, 51]. In these studies, Ca-HMB proved very effective for reducing the strong catabolic effect of HS in plantar flexor muscles and enhancing plantar flexor muscle recovery after reloading [60].

We have not previously examined the effect of Ca-HMB on the dorsiflexor muscle group in HS or R. The extensor digitorum longus (EDL) muscle is composed primarily of fast-twitch (type II) fibers [61] and like fast-contracting plantar flexor muscles, it has a decrease in muscle function with aging to a greater extent than postural slow-twitch (type I) fiber types such as those in the soleus muscle [62–65]. However, unlike plantar flexors, EDL is primarily used to assist the tibialis anterior muscle in lifting and positioning the foot during locomotion. We expected that dorsiflexor muscles would not be affected as negatively by unloading as plantar flexors because the dorsiflexors would be able to continue to contract and lift the weight of the foot during HS. This contrasts to plantar flexors which would be unable to contract against the body weight of the animal during HS. Consistent with this idea, we have observed little or no atrophy in response to HS in the EDL muscle (unpublished observations). Similarly, other studies have also reported that the rat EDL does not undergo atrophy when subjected to HS [66–72]; however, unlike HS, immobilization by casting has been reported to induce marked atrophy in the EDL muscle [73–75].

It was not known if Ca-HMB treatment would improve EDL muscle mass or function in response to HS or R in aged rats, if HS did not induce a significant loss of muscle mass, and if R did not induce EDL hypertrophy. Therefore, in this study, we were interested in evaluating the effects of Ca-HMB treatment on the EDL muscle. We expected that this model would not have acute atrophy during HS yet would have reduced loading. However, we recognize that studying EDL during HS was complex and this muscle could also undergo atrophy during HS because some studies have reported a modest atrophy in the EDL with limb unloading [36, 76–79].

## 2. Materials and Methods

### 2.1. Animal Care

In the current study, we used a research design that was similar to our previous two studies using Ca-HMB [43, 51] which had shown Ca-HMB-induced benefits in plantar flexor muscles after unloading and reloading. In this study, we also used sixty-four male Fisher 344 × Brown Norway rats (34-36 months of age) that were obtained from Harlan Sprague Dawley (Indianapolis, IN) which houses the National Institute on Aging colony. The animal care standards followed the guidelines as published by the U.S. Department of Health and Human Services and as described in the Animal Welfare Act (PL89-544, PL91-979, and PL94-279). These care standards were consistent with the American Association for Accreditation of Laboratory Animal Care (AAALAC) and the Guide for the Care and Use of Laboratory Animals. All experimental procedures were approved by the Institutional Animal Care and Use Committee from West Virginia University and the University of Tennessee Health Science Center. The University of Tennessee Health Science Center maintains an AAALAC-certified animal care facility.

### 2.2. Hindlimb Suspension (HS) and Reloading (R) after HS

We used the same research design as our previous two studies using Ca-HMB [43, 51]. All of the control and experimental animals were singly housed in custom-made cages with a front door as an entry point for easy access to the animals for care and gavage. The aged rats were randomly placed into a control group (C, *n* = 32) that maintained normal cage activity; a bilateral hindlimb-suspended group (HS, *n* = 16), where both hindlimbs were raised from the cage floor for 14 days; or a reloaded group (R, *n* = 16), which received 14 days of unloading; then, the animals were returned to normal cage activity for 14 days. Hindlimb suspension was performed as previously described in multiple studies [24, 37, 40, 42, 43, 45, 51, 60]. Briefly, the wire tail harness was attached to the proximal one-third of the tail then attached to a swivel that permitted 360° of movement of the rat around the cage while raising the hindlimbs from the floor. The foot was not immobilized, and it was free to move but remained in the HS position unloaded. The rats were able to eat and drink ad libitum over the course of the study. The tail harness was removed after 14 days of HS for rats in the R group, and the animals were returned to their cages to resume their normal loading of their hindlimbs, ambulation, and movement around the cage. All animals were singly housed.

### 2.3. Ca-HMB Treatment

HMB is available in both calcium (Ca-HMB) salt or free acid (FA-HMB) forms, and both Ca-HMB [59, 60] and FA-HMB [80] have been reported to improve the anabolic nature of muscle in older humans. We chose to use Ca-HMB because we and others have previously found that this form of HMB improved muscle remodeling in the plantar flexors of old rats after HS [43, 51]. The animals received either 170 mg of Ca-HMB or distilled water by gavage feeding. To ensure that HMB was systemically available to the animals prior to limb unloading, we began the Ca-HMB or water placebo gavages seven days prior to HS. To do this, the animals were held in a head-up position, while the neck and head were supported. This mimicked the same position that we would use to gavage the animals for the HS loading period. This pre-HS period also provided a conditioning experience for the animals so they would be accustomed to the gavage approach prior to the animals being placed in HS [43, 51]. We continued daily gavages with the animals kept in the tail suspension harness. To do this and to prevent the hindlimbs being loaded during gavage, the cage door of the custom HS cages was opened to gain access to the rat. The back and shoulders of the rat were supported by the investigator, then the animal was raised from the head-down to the head-up position, with the head and neck supported in one hand. The tail harness was intact but rather than supporting the animal's body weight, the investigator supported the animal during the gavage. The feet of the rat never touched the cage floor during the gavage. The blunt gavage needle was then inserted into the mouth and pharynx of the rat. The contents were given to the rat and after a full volume of water or Ca-HMB, the rat was returned to the head-down position (with the tail resuming support of the hind limbs) and the cage door closed.

### 2.4. Research Design

**Figure 1:**
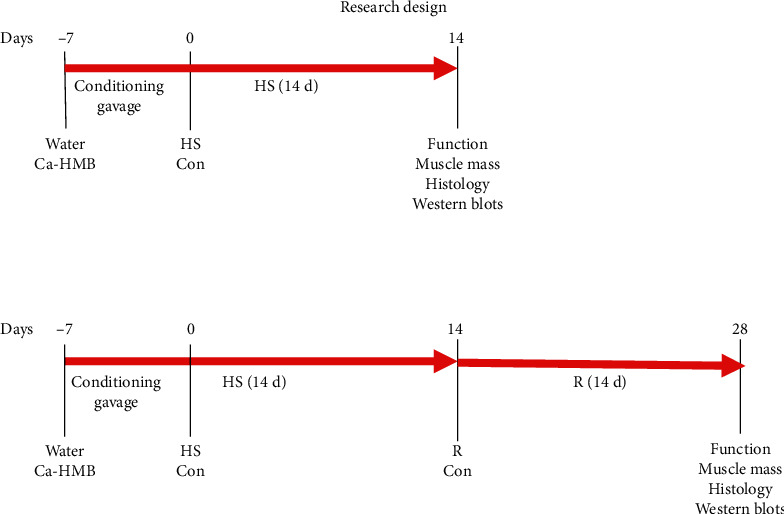
Research design. Animals were divided randomly into two groups that received water or Ca-HMB by gavage for 7 days (day -7 to day 0) prior to experimental intervention. This provided a 7-day conditioning period for the animals to accommodate the gavage. Animals then remained as controls in their cage or were placed in hindlimb suspension (HS) to unload their hindlimbs for 14 days. Some animals were euthanized after 14 days of HS. Other animals were released from the HS and allowed to ambulate normally for 14 days of recovery (R). Control animals for the R group remained fully ambulated throughout the entire 35-day experimental period.

Muscle data was obtained from both the Ca-HMB and water-gavaged animals. The research design for the study is shown in Figure 1. It was similar to our other studies with HMB and other nutraceuticals [40, 41, 43, 51]. Two groups of ambulatory nonsuspended control animals were used. Eight control animals for the HS group (HS Con) and a second control group of eight were used as controls for the animals that received HS for 14 days, followed by 14 days of reloading (R Con). Eight animals were examined in each group after 14 days of HS, and eight animals were examined after 14 days of HS, followed by 14 days of recovery.

### 2.5. *Ex Vitro* EDL Contractile Responses

*Ex vitro* isometric muscle contractile properties were examined in the extensor digitorum longus (EDL) muscles of control and Ca-HMB-treated rats. The muscles were placed in an oxygenated Ringer's solution at 20°C. The muscles were stimulated with a constant current stimulator (Aurora Scientific Aurora Ontario, Canada) with a 200 *μ*s pulse width, and muscle force was measured with a 300C dynamometer (Aurora Scientific, Aurora Ontario, Canada) [81, 82]. Muscles were adjusted to the optimal muscle length (*L*_o_), then peak isometric tetanic force (*P*_o_) was obtained at 10, 20, 40, 50, 75, and 100 Hz, with 3 minutes of rest between each contraction. EDL twitch responses were obtained for peak isometric twitch force (PT), peak twitch contraction tension (CT), and 1/2 relaxation time of twitch contraction ((1/2)RT) as previously described [37, 38, 40, 81–84]. After resting, muscle fatigue was assessed by stimulating the muscle at 40 Hz for 3 minutes with 666 ms of rest between each contraction that lasted 333 ms [37, 82, 85, 86]. The fatigue index was calculated as the difference in force from the initial three contractions to the mean of the final three contractions [38, 82].

### 2.6. EDL Weight and Tissue Preparation

At the end of the experimental period, and with the animals deeply anesthetized, the EDL muscles were removed from both limbs, blotted, and then weighed. Once complete, the animals were immediately euthanized by removing the heart. A block obtained from the midbelly of the EDL muscle was frozen in liquid nitrogen-cooled isopentane and stored at -80°C until subsequent analyses.

### 2.7. Identification of EDL TUNEL-Positive Apoptotic Nuclei

EDL muscle tissue cross sections were labeled with a terminal dUTP nick-end (TUNEL) labeling assay according to the manufacturer's recommendations (11684795910; Roche Applied Science, Indianapolis, IN). Thereafter, the EDL tissue sections were fixed with cold 4% paraformaldehyde in phosphate-buffered saline (PBS) and incubated overnight at 4°C in a rat anti-lamina monoclonal antibody (MAB 1914, Millipore, Billerica, MA). The tissue sections were incubated with the appropriate secondary antibody (712-025-150, Jackson ImmunoResearch Laboratories, West Grove, PA) to visualize the boundaries of the fiber (lamina), along with the TUNEL-positive nuclei. The sections were mounted with 4′,6-diamidino-2-phenylindole (DAPI; ProLong™ Gold Antifade Mountant, Fisher Scientific). The sections were viewed, and the images captured with a Zeiss LSM 510 Meta confocal microscope (Carl Zeiss Microimaging Inc., Thornwood, NY) in the institutional imaging core facility. The number of TUNEL- and DAPI-positive nuclei (satellite cells + myonuclei) that were beneath the basal lamina were quantified from ~1000 fibers/EDL muscle section. Data were expressed as an apoptotic index, which was calculated by counting the number of TUNEL-positive nuclei divided by the total number of nuclei (i.e., DAPI-positive nuclei). No distinction was made between satellite cell and myonuclei, and all muscle nuclei were pooled for analysis. These approaches are similar to previous published methods that are established in our lab [43, 47, 87, 88].

### 2.8. Fiber Morphology

The average muscle fiber cross-sectional area (CSA) was obtained by tracing 750-1200 fibers in each EDL tissue section using approaches established in our laboratory [38, 40, 41, 51, 84, 89]. EDL muscle fiber CSA was calculated by the ImageJ software (NIH).

### 2.9. Western Blots from EDL Lysates

Approximately 75-100 *μ*g of muscle was homogenized in RIPA buffer containing protease inhibitors (P8340; Sigma-Aldrich, St. Louis, MO) and phosphatase inhibitors (P2850 and P5726; Sigma-Aldrich) using approaches published in our lab [39, 82, 85, 90]. The protein content of the muscle lysates was measured using the DC Protein Assay kit (500-0116; Bio-Rad, Hercules, CA). Forty micrograms of protein was separated by sodium dodecyl sulfate-polyacrylamide gel electrophoresis (SDS-PAGE) on a 4-12% gradient polyacrylamide gel (NP0335BOX; Invitrogen, Carlsbad, CA) using previously published protocols from our laboratory [38, 39, 82, 85, 90]. Western blots were incubated at 4°C for 12 h with primary antibodies directed against Bax (#2772, Cell Signaling Technology), cleaved caspase-3 (#9664, Cell Signaling Technology), cleaved caspase-9 (#9509, Cell Signaling Technology), and Bcl-2 (#2876, Cell Signaling Technology, Boston, MA). The signals were developed using a chemiluminescent substrate (Lumigen TMA-6; Lumigen, Southfield, MI), and the protein bands were assessed as optical density × band area and expressed in arbitrary units using ImageJ (NIH).

### 2.10. Statistical Analysis

The results are reported as means ± SD. Differences in means between groups were determined by multiple analysis of variance (MANOVA) Hotelling's *T*-square test. Bonferroni's post hoc analyses were performed between significant means. A *p* ≤ 0.05 was considered to represent statistically significant mean differences.

## 3. Results

### 3.1. Body Weight

**Figure 2:**
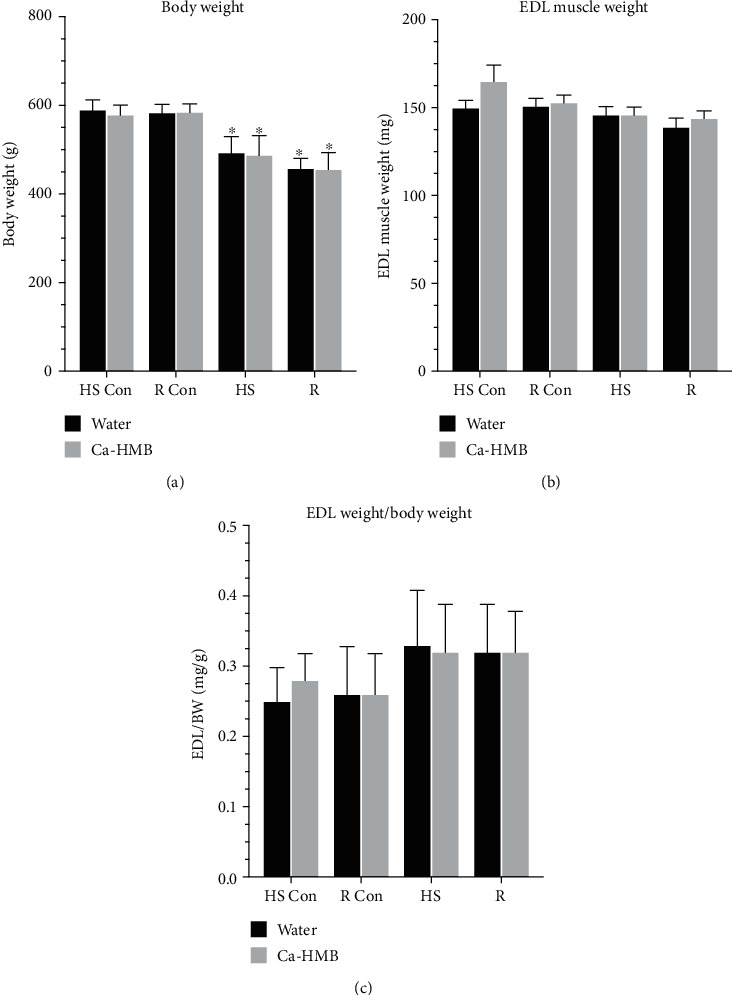
Muscle and bodyweight. (a) The body weight of water-gavaged and Ca-HMB-gavaged animals are shown after 14 days of hindlimb suspension (HS) or 14 days of HS followed by 14 days of reloading (R). The animals received Ca-HMB or the vehicle (water) daily by gavage, for a total of 21 days (HS Con and HS) or for 32 days (R Con and R). (b) EDL muscle wet weight was obtained in cage control animals for the hindlimb suspension group (HS Con), cage control animals for the recovery group (R Con), and water-treated and Ca-HMB-treated animals after 14 days of hindlimb suspension (HS) or after 14 days of hindlimb suspension followed by 14 days of reloading (R). (c) The ratio of EDL muscle wet weight to body weight was reported. A MANOVA followed by Bonferroni's post hoc analyses were used to evaluate the differences between the group means. The data are expressed as mean ± SD. ^∗^*P* < 0.05; HS or R vs. control animals for that experimental condition.

The body weights of all the experimental animals did not differ at the beginning of the study. The body weights of nonsuspended, water- and Ca-HMB-treated animals were reduced by ~4% and ~5%, respectively, over the course of the study, relative to the first day of the experimental period. This likely represented a general aging-associated loss of body weight. In general, 14 days of HS significantly lowered the animals' body weight by ~18% in water-gavaged and by ~17% in the Ca-HMB- and water-gavaged groups (Figure 2(a)). The body weights in both of these groups continued to decline during the fourteen-day recovery period following the HS protocol so that the animals' body weights were ~21.6% and ~22.1% lower in water-gavaged and Ca-HMB-gavaged animals following the R period, relative to the starting body weight. This suggests that Ca-HMB did not slow the loss of body weight due to aging or HS.

### 3.2. Muscle Wet Weight

EDL muscle wet weight was not different in control muscles from water-treated and Ca-HMB-treated animals. As expected from our pilot studies, HS did not significantly reduce EDL muscle wet weight in either group, nor did reloading significantly alter muscle weight in water-treated or Ca-HMB-treated animals (Figure 2(b)). The ratio of EDL muscle wet weight to the animals' body weight was not significantly different at any time point (Figure 2(c)).

### 3.3. EDL *Ex Vitro* Physiology

**Figure 3:**
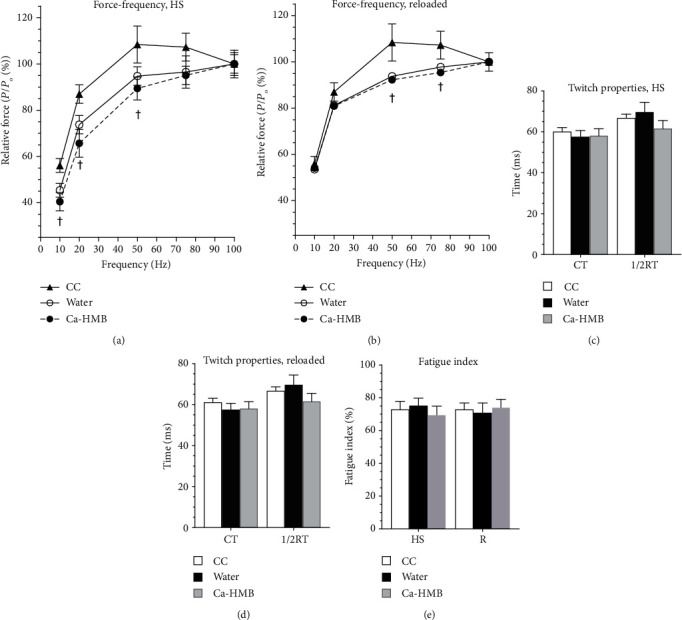
Muscle function. The force-frequency response of the EDL was obtained *ex vivo* (a) after 14 days of hindlimb suspension (HS) and (b) after 14 days of hindlimb suspension followed by 14 days of reloading. The animals received either Ca-HMB or water daily for 7 days before and throughout the experimental period and compared to cage control animals (CC) that were gavaged with the vehicle. A MANOVA followed by Bonferroni's post hoc analyses were used to evaluate the differences between the group means. ^†^*p* < 0.05; water and Ca-HMB vs. CC (cage control). (c) Twitch contraction time (CT) and one-half relaxation time ((1/2)RT) were analyzed in cage control animals gavaged with water or Ca-HMB rats after 14 days of HS or (d) after 14 days of hindlimb suspension followed by 14 days of reloading. Data are presented as mean ± SD. (e) A modified Burke protocol was implemented to assess muscle fatigue in the EDL. The data are presented as mean ± SD as a measure of the fatigue index, calculated as a percent change from the first to the last contraction (120th). The groups were cage control animals gavaged with water or Ca-HMB after 14 days of HS or after 14 days of hindlimb suspension followed by 14 days of R.

Isometric contractile properties of EDL from Ca-HMB-gavaged and water-gavaged HS and R animals were measured and compared to the contractile responses from a cage control group [81, 84, 91]. The force-frequency curve of the HS group was neither shifted to the right nor left, but absolute force from EDL muscles of the Ca-HMB-treated and water-treated animals had significantly lower forces at 10, 20, and 50 Hz as compared to the cage control animals after HS. The relative forces were depressed only at 50 and 75 Hz in both Ca-HMB-treated and water-treated rats of the R group as compared to control animals (Figure 3). This suggests that Ca-HMB did not prevent the depression in force production from either HS or reloading.

Consistent with the data from the force-frequency curve, the twitch contraction time (CT) time was similar between the Ca-HMB-treated and water-treated animals, in both the HS and R groups. However, the Ca-HMB-treated animals had a similar lower (1/2)RT as compared to the water-treated animals (61.6 ± 15.1 ms vs. 79.7 ± 17.6 ms) after HS. In the R group, Ca-HMB did not affect either CT or (1/2)RT compared to the respective water-treated or cage control animals (Figures 3(c) and 3(d)). Finally, Ca-HMB did not alter the fatigue index in either the HS or R groups as compared to the water-treated HS animals, and there were no differences between HS or R groups for either Ca-HMB or water placebo treatment (Figure 3(e)).

### 3.4. Apoptotic Signaling Proteins in the EDL

**Figure 4:**
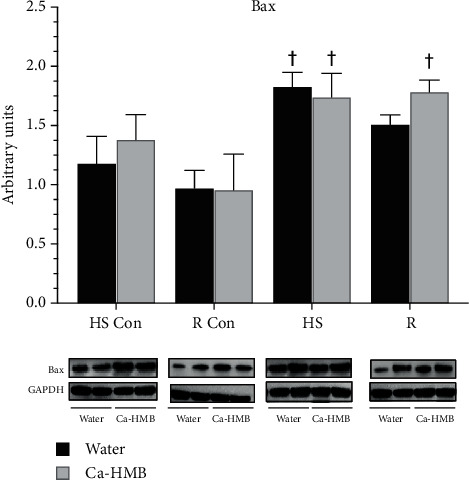
Bax. Bax protein abundance was determined by western blot analysis in the EDL muscles of rats under control, hindlimb suspension, or reloading conditions. The groups include controls for the hindlimb suspension group (HS Con), controls for the recovery group (R Con), and in experimental animals after 14 days of hindlimb suspension (HS) or after 14 days of hindlimb suspension followed by 14 days of reloading (R). The animals received Ca-HMB or the vehicle (water) daily by gavage, for a total of 21 days (HS Con and HS) or for 32 days (R Con and R). Eight animals were in each experimental group. GAPDH was used as a loading control. The data were normalized to GAPDH, and they are shown as mean ± SD. A MANOVA followed by Bonferroni's post hoc analyses were used to evaluate the differences between the group means, ^†^*p* < 0.05, HS or R vs. control animals for that experimental condition.

We have previously found an increase in apoptotic proteins and signaling in plantar flexor muscles subjected to HS or R [29, 37, 40, 43, 46, 92], although the apoptotic signaling response of the dorsiflexor EDL muscle was not known. We found that the protein abundance of the proapoptotic Bax protein was elevated in the EDL muscle after HS and R conditions, but there was no difference between water-treated and Ca-HMB-treated EDL muscles (Figure 4).

**Figure 5:**
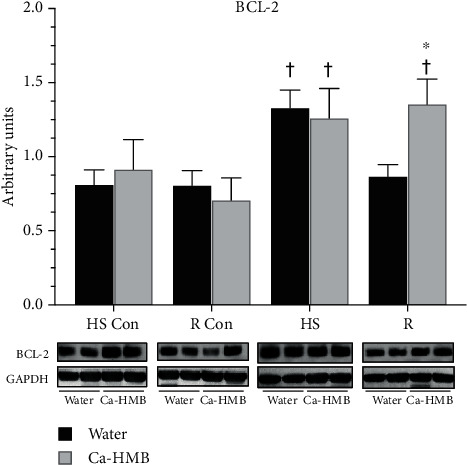
BCL-2. BCL-2 protein abundance was determined by western blot analysis in the EDL muscles of rats under control, hindlimb suspension, or reloading conditions. The groups include controls for the hindlimb suspension group (HS Con), controls for the recovery group (R Con), and in experimental animals after 14 days of hindlimb suspension (HS) or after 14 days of hindlimb suspension followed by 14 days of reloading (R). The animals received Ca-HMB or water daily by gavage, for a total of 21 days (HS Con and HS) or for 32 days (R Con and R). Eight animals were in each diet and experimental group. GAPDH was used as a loading control. The data were normalized to GAPDH and were expressed as mean ± SD. A MANOVA followed by Bonferroni's post hoc analyses were used to evaluate the differences between the group means. ^†^*p* < 0.05, HS or R vs. control animals for that experimental condition. ^∗^*P* < 0.05, water vs. Ca-HMB within the same condition;

The antiapoptotic BCL-2 protein was elevated after HS in both vehicle- and Ca-HMB-treated EDL muscles. In the reloaded muscles, BCL-2 had returned to control levels in the water-gavaged animals, but it was still elevated in the EDL muscles of animals treated with Ca-HMB (Figure 5).

**Figure 6:**
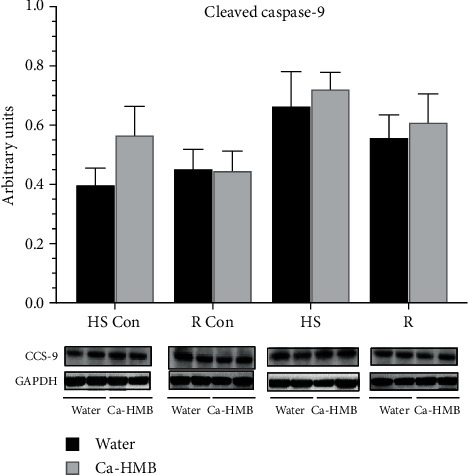
Cleaved caspase-9. Cleaved caspase-9 protein abundance was determined by western blots in the EDL muscles of rats under control, HS, or R conditions. The groups include controls for the hindlimb suspension group (HS Con), controls for the reloading group (R Con), and in experimental animals after 14 days of hindlimb suspension (HS) or after 14 days of hindlimb suspension followed by 14 days of reloading (R). The animals received Ca-HMB or water daily by gavage. Eight animals were in each group. The data were normalized to GAPDH and were expressed as mean ± SD. A MANOVA followed by Bonferroni's post hoc analyses were used to evaluate the differences between the group means.

**Figure 7:**
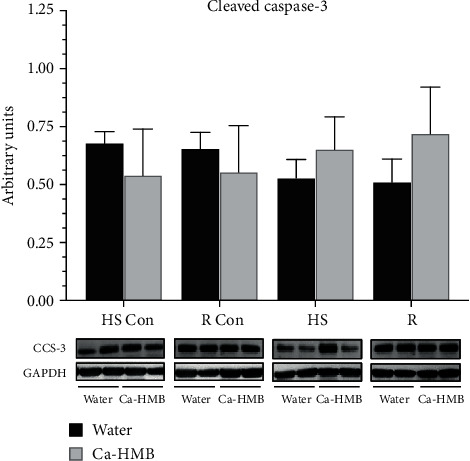
Cleaved caspase-3. Cleaved caspase-3 protein abundance was determined by western blot analysis in the EDL muscles of rats under control, hindlimb suspension, or reloading conditions. The groups include controls for the hindlimb suspension group (HS Con), controls for the recovery group (R Con), and in experimental animals after 14 days of hindlimb suspension (HS) or after 14 days of hindlimb suspension followed by 14 days of reloading (R). The animals received Ca-HMB or water daily by gavage. Eight animals were in each group. The data were normalized to GAPDH and were expressed as mean ± SD. A MANOVA followed by Bonferroni's post hoc analyses were used to evaluate the differences between the group means. CCS-3: cleaved caspase-3.

Despite HS-induced increases in Bax in the EDL, neither cleaved caspase-9 protein abundance (Figure 6) or cleaved caspase-3 (Figure 7) were elevated in the EDL muscle after HS or R, and there were no differences between EDL muscles in the Ca-HMB-gavaged and water-gavaged animals.

### 3.5. TUNEL Labeling in EDL Muscles

**Figure 8:**
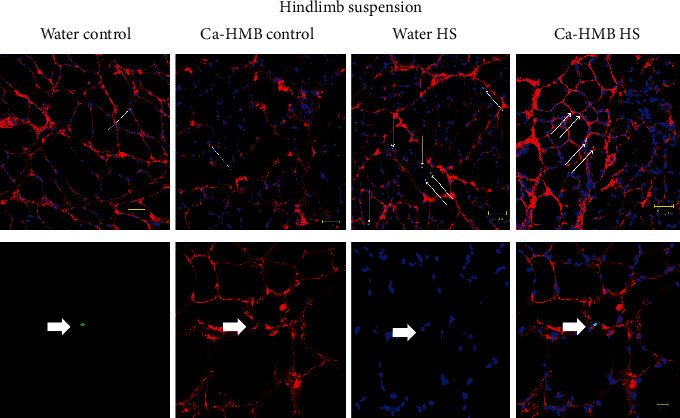
Apoptotic nuclei after hindlimb suspension. Top row: representative tissue sections from the EDL muscle, with fluorescent staining for TUNEL (green) to identify apoptotic nuclei in control and hindlimb-suspended muscles. DAPI identified all nuclei (blue). The basal lamina (red) was identified to confirm that the TUNEL-positive nuclei were myonuclei. The conditions were water control (Water control), Ca-HMB control (Ca-HMB control) of HS (Water HS), and Ca-HMB after 14 days of HS (Ca-HMB HS). The arrows show TUNEL-positive nuclei lying below or immediately adjacent to the basal lamina of the muscle fibers. Bottom row: a higher magnification showing the individual markers for TUNEL, laminin, DAPI, and the combined images. The arrows show TUNEL-positive nuclei lying below or immediately adjacent to the basal lamina of the muscle fibers.

**Figure 9:**
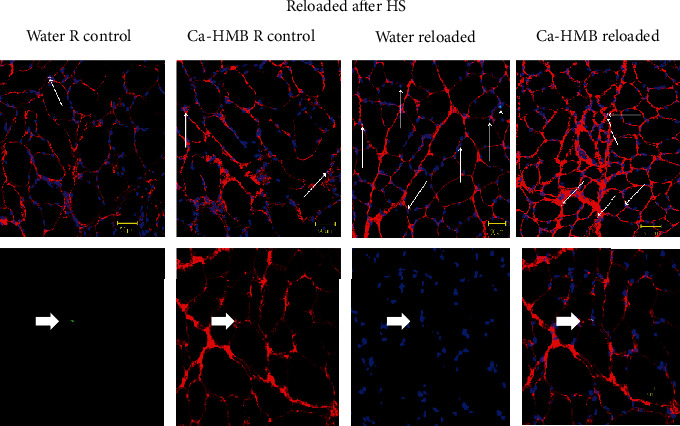
Apoptotic nuclei after recovery. Top row: representative tissue sections from the soleus muscle, with fluorescent staining for TUNEL (green) to identify apoptotic nuclei in control muscles and muscles after 14 days of hindlimb suspension followed by 14 days of reloading (R). DAPI identified all nuclei (blue). The basal lamina (red) was identified to confirm that the TUNEL-positive nuclei were myonuclei. The conditions were vehicle control for reloaded animals (Water R control), Ca-HMB control for reloaded animals (Ca-HMB con), vehicle reloaded after 14 days of HS and 14 days of reloading (Vehicle reloaded), and Ca-HMB after 14 days of HS followed by 14 days of reloading (Ca-HMB reloaded). The arrows show TUNEL-positive nuclei lying below or immediately adjacent to the basal lamina of the muscle fibers. Bottom row: a higher magnification showing the individual markers for TUNEL, laminin, DAPI, and the combined images. The arrows show TUNEL-positive nuclei lying below or immediately adjacent to the basal lamina of the muscle fibers.

Nuclear DNA breaks in the pool of satellite cells and myonuclei in the EDL muscles were measured by TUNEL labeling and reported as an apoptotic index (the percent of TUNEL-positive myonuclei to the total number of myonuclei). There was very little evidence of nuclear apoptosis (in all myonuclei and satellite cells) in EDL muscles from the control animals. However, TUNEL-positive nuclei as determined from the total nuclei pool of myonuclei and satellite cell nuclei, increased in EDL muscles from HS (Figure 8) and R (Figure 9) animals.

**Figure 10:**
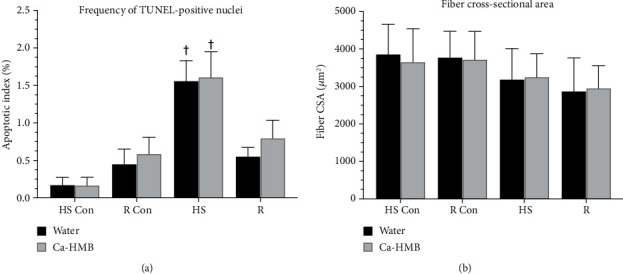
Apoptotic index and muscle fiber size. (a) The apoptotic index was calculated from tissue cross sections of the EDL muscle by determining the number ratio of TUNEL-positive nuclei to total nuclei in EDL cross sections from muscles of control animals for the hindlimb suspension group (HS Con), the recovery group (R Con), and in experimental animals after 14 days of hindlimb suspension (HS) or after 14 days of hindlimb suspension followed by 14 days of reloading (R). Only nuclei that were directly below or touching the basal lamina were counted. Therefore, this evaluation included both satellite cell and myonuclear cell populations. The animals received either Ca-HMB or water daily by gavage. A MANOVA followed by Bonferroni's post hoc analyses were used to evaluate the differences between the group means; ^†^*p* < 0.05, HS or R vs. control animals for that experimental condition. (b) Fiber cross-sectional area (CSA) was obtained by planimetry in the EDL muscles of control animals for the hindlimb suspension group (HS Con), the recovery group (R Con), and in experimental animals after 14 days of hindlimb suspension (HS), or after 14 days of hindlimb suspension followed by 14 days of reloading (R). The animals received Ca-HMB or water daily by gavage. A MANOVA followed by Bonferroni's post hoc analyses were used to evaluate the differences between the group means. The data are presented as mean ± SD.

While there was no significant difference between the Ca-HMB-treated or water-treated HS animals, HS significantly increased the percentage of TUNEL-positive nuclei as compared to their respective control group (1.61 ± 0.8% vs. 0.17 ± 0.2%), but there was no significant difference between water-treated and Ca-HMB-treated EDL muscles (Figure 10(a)). The apoptotic index which included myonuclei and satellite cells was similar in EDL muscles from control and R groups, and there was no difference between the Ca-HMB-treated and water-treated EDL muscles.

### 3.6. EDL Fiber Cross-Sectional Area (CSA)

The cross-sectional area (CSA) of the EDL muscle fibers were measured in the HS and R groups after water- or Ca-HMB treatment. Neither HS nor R in either water-treated nor Ca-HMB-treated animals had no significant changes in EDL fiber CSA as compared to the EDL muscles from control animals in response to HS or R. These data suggest that Ca-HMB had no effect on hypertrophy or atrophy in EDL under conditions of HS or R (Figure 10(b)).

## 4. Discussion

HMB has been shown to reduce protein degradation [93], prevent muscle atrophy, or restore lost appendicular muscle mass in older people with reduced lean body mass [59, 60, 80, 94–96]. HMB has also been reported to improve muscle mass and function and increase muscle mitochondria biogenesis [93] in response to muscle wasting and acute unloading and reloading in aging [43, 51, 60]. However, the benefits of HMB have not been universally found in humans [80] and the benefits of HMB-induced increased lipid storage in the vastus lateralis fibers of older persons after bedrest are unclear [97].

The Ca-HMB form of HMB has been proposed to affect muscle function and size via various mechanisms, including decreasing protein degradation [98, 99], reducing signaling via the proteasome-ubiquitin degradation pathway [99, 100], increasing muscle protein synthesis [7, 100], reducing apoptosis [43, 51, 62], and promoting the proliferation and differentiation of myoblasts [2]. Ca-HMB is also thought to be metabolized into HMB-CoA, which would provide a carbon source for cholesterol synthesis and improve muscle cell growth [95, 101–104]. When combined with a hypertrophic stimulus such as exercise or loading, Ca-HMB has also been shown to reduce muscle damage, as evaluated by lower creatine kinase (CK) and lactate dehydrogenase [101, 105, 106], and this should help muscle recover from reloading-induced damage after a period of unloading or disuse.

In this study, we sought to determine if Ca-HMB supplementation would improve muscle mass or function in dorsiflexor muscles from old rats, where the limb was unloaded but the EDL muscle did not become severely atrophic. We chose to evaluate the EDL muscle because our lab and others (although not all labs) have failed to find a loss of EDL muscle mass after HS. In our model of HS, EDL is unloaded but the foot is not restrained. Therefore, the dorsiflexors are able to contract and lift the foot in the suspended position. In our hands, EDL was not significantly atrophied by HS or R because muscle mass and function were not lower in old rats after HS or R as compared to old cage control rats. Unlike the plantar flexors which have significant losses of muscle mass with HS [29, 37, 40, 41, 43, 46] that can be partially prevented by Ca-HMB treatment [43, 51, 62], in this study as compared to water treatment, Ca-HMB had no effect on muscle mass or function in either HS or R conditions. Although HS and R increased Bax and BCL-2 apoptotic signaling proteins in the EDL muscle, Ca-HMB treatment did not suppress apoptotic signaling in EDL muscles of old rats as compared to water treatment.

### 4.1. Body Weight and EDL Muscle Mass after HS

HS causes significant systemic and muscle-specific changes including altered hypodynamic cardiovascular control [23], reduced bone metabolism [5, 42], delayed wound healing [107], increased oxidative stress [24, 58, 108, 109], and elevated signaling for apoptosis [10, 37, 40, 41, 43, 56, 62, 110]. However, in the current study, while the body weights of all the animals subjected to HS were reduced, Ca-HMB introduced by gavage was unable to significantly attenuate these losses as compared to water treatment by gavage.

We chose to study the EDL muscle in this study, because in our hands we had previously observed that the EDL muscle did not lose appreciable weight during HS (unpublished results) and this was confirmed in the current study. Furthermore, when the EDL muscle weights were normalized to body weight, there were no significant differences in these normalized muscle weights either between or within any of the groups of animals as all animals lost body weight (Figure 1). This is unlike the well-documented loss of muscle mass by HS that we and others have found in both fast gastrocnemius and plantaris and slow-contracting soleus plantar flexor muscles of old rodents [37–43, 45, 46, 48–50, 92].

The data in our study are consistent with other studies that also found that the rat EDL does not undergo atrophy when subjected to HS [66–72]. However, this is not a universal observation because other studies have reported a modest but significant atrophy in EDL but not as much as the loss found in the soleus in response to HS [36, 76–79]. In contrast to HS, immobilization-induced disuse such as casting has shown a more marked atrophy in the EDL muscle [73–75]. Likely, this lack of extensive EDL muscle loss in our HS model occurred because the dorsiflexor muscles maintain high EMG activity during HS [72]. In addition, HS does not immobilize the foot so that the dorsiflexors can still contract and dorsiflex the foot during HS with a load that is similar to normal ambulation. Another important point is that the body position in HS and in all non-weight-bearing states is a plantar flexor posture with the toes generally pointing towards the bottom of the cage. However, this plantarflexion position would provide some mild stretch of the EDL during HS which would be expected to offset muscle loss from disuse. In support of this idea, it is known that stretching will attenuate muscle loss with limb immobilization [111, 112]. Furthermore, large degrees of muscle stretching and lengthening ~10-20% beyond a resting length provides a very powerful anabolic stimulus to prevent atrophy and induce growth [113–118] even under conditions of denervation where EMG activity is eliminated [119, 120]. EDL stretching may have been sufficient to minimize catabolic signaling that would have occurred as a result of muscle disuse in our model of HS, although the degree of stretching of EDL was not sufficient to induce hypertrophy beyond its basal level. Thus, the maintenance of EMG activity, the ability to lift the foot during HS, and the mild stretching of EDL during HS, probably together prevented a significant loss of EDL muscle mass. This is very different from the plantar flexor muscles that do not lift the animal's body weight like normal locomotion during HS. Furthermore, the soleus muscle, for example, has a rapid and marked decrease in EMG activity to near zero levels during HS and resumes lifting the animal's body weight during R [112, 121, 122]. In the posture that is assumed during HS, the ankle of the rat moves into plantarflexion which shortens and unloads the triceps surae muscles and results in high degrees of muscle loss, especially in the slow-contracting soleus muscle [49, 123]. Thus, unlike EDL, muscles in the plantar flexor group experience atrophy during HS, as they would both have reduced EMG activity, no ability to contract by lifting the animal's body weight, and be primarily in a shortened position during HS [37, 38, 40, 41, 51, 124].

It is not that EDL is unable to atrophy because there was some mild decrease in fiber size in EDL as compared to control muscles after HS or R, but this was insufficient to impact total EDL muscle wet weight. Thus, a different model such as immobilization would have been expected to induce a significant loss of muscle mass in EDL. In support of this idea, Du et al. [125] have found that fixing the ankle in dorsiflexion, which shortens the EDL muscle during HS, results in significant atrophy of EDL in rats in a similar fashion as seen in the gastrocnemius, soleus, and plantaris of the rat during HS, which also shortens and atrophies these muscles. Although speculative, we hypothesize that Ca-HMB may have slowed atrophy in EDL like it does in the plantar flexors during unloading, if a different model such as immobilization restraint was used to induce significant atrophy in the EDL muscle, rather than HS which does not induce significant EDL muscle atrophy in aged rats, at least in our hands.

### 4.2. EDL Muscle Force and Fatigue

HMB has been shown to improve strength and fatigue resistance in quadriceps muscles of humans including the elderly in multiple studies especially when HMB treatment was combined with exercise [53, 95, 103, 126–130]. However, we did not observe an increase in muscle force or fatigue resistance in the EDL muscles of Ca-HMB-treated rats after unloading by HS or during reloading as compared to rats that received water.

Although unloading or reloading after HS did not make a marked change in the maximal force production of EDL, there was a depression of force production at several points along the force-frequency curve in EDL muscles after both HS and R. Furthermore, Ca-HMB had no effect on the force-frequency relationship of EDL in either HS or R conditions (Figures 3(a) and 3(b)). In addition, Ca-HMB showed no improvements in CT, (1/2)RT (Figures 3(c) and 3(d)), or the fatigue index (Figure 3(e)) of EDL muscles in either the HS or the R groups. This may be explained, at least in part, by the fact that this HS protocol did not cause extensive muscle wasting in the EDL muscle so there was no large decrement to overcome, and reloading had an insufficient hypertrophic stimulus to enhance protein synthesis. In a study by Hoffman et al. [107], it was suggested that Ca-HMB supplementation may produce the greatest benefits when muscle damage is increased, which has been shown to be higher in untrained individuals than in trained persons [131, 132]. Furthermore, HMB has been shown to improve EDL and soleus muscle protein synthesis in muscle wasting that was induced by partial hepatectomy in rats [133]. In addition, adding Ca-HMB to a hypertrophic stimulus (e.g., exercise or loading) and/or other anabolic nutraceuticals has been shown to improve the hypertrophic response of plantar flexor muscles in rodents [80, 134, 135] or quadriceps muscles in humans [53]. However, Ca-HMB treatment to aged rats without an exercise intervention did not improve gastrocnemius mass or function [136]. While studies that showed improvements in strength in humans were conducted in individuals with varied training experiences, those that have been performed solely on trained individuals have produced mixed results, with the majority [100, 137–140] showing Ca-HMB to be ineffective at improving strength. This is likely because trained muscles had adapted to the increased loading and therefore did not have a large anabolic stimulus to grow further. However, Ca-HMB has been shown to improve quadriceps muscle strength when combined with the anabolic stimulus of exercise in older humans [59, 80, 141]. These published studies and our current data support the idea that Ca-HMB supplementation has the greatest anabolic benefits to increase mass and strength or reduce muscle wasting when the muscle has a major insult (e.g., damage or loading to induce repair and/or muscle growth after severe atrophy). It is likely that in our model of reloading after HS, EDL did not have a strong anabolic stimulus for improving muscle mass and muscle force capacity because EMG activity was likely not decreased during HS, and during reloading, EDL likely had a similar contractile effort to raise the foot during cage ambulation, as when the foot was lifted during HS. Nevertheless, it is not clear if an anabolic signal combined with HMB in old EDL muscles would have enhanced protein synthesis beyond the increase that would be expected from exercise or loading alone in this muscle. This is suggested by recent data that show that HMB supplementation in the quadriceps of free-living healthy older men did not enhance muscle strength or mass greater than that of resistance training alone [80]. Furthermore, other data indicate that no additional benefit for improving rat gastrocnemius and soleus plantar muscle mass or function was obtained by Ca-HMB treatment as compared to resistance exercise alone [142].

### 4.3. Death Signaling in the EDL

Nuclear apoptosis and increased apoptotic signaling have been shown to be increased in atrophied plantar flexor muscles of old rats after hindlimb unweighting [1, 10, 11, 22, 37, 62, 110, 143–148]. However, it was not known if apoptosis or apoptotic signaling precedes atrophy or was in response to it. Ca-HMB has been shown to exert positive effects in suppressing the abundance of apoptotic proteins in plantar flexor muscles during HS or R [43, 98, 149]. However, in the present study, Ca-HMB had no effect on changing the abundance of proapoptotic cleaved caspase-3 or cleaved caspase-9 proteins in EDL after HS or R. Interestingly, the amounts of the proapoptotic Bax protein was significantly higher in the EDL muscles from the Ca-HMB-treated R group as compared to the vehicle-treated animals, yet this was counterbalanced by the increase in the antiapoptotic protein BCL-2 in the Ca-HMB-treated R animals as compared to the respective control animals.

We were not interested in separating apoptosis signaling in postmitotic myonuclei which carry the primary responsibility of maintaining cell homeostasis and in satellite cell nuclei which can divide and contribute to or maintain the myonuclear pool for growth and recovery postinjury or after loading. Therefore, we quantified apoptotic signaling in all muscle nuclei (including satellite cells + myonuclei) by western blot analysis and all nuclei that were TUNEL positive that resided below the basal lamina. In this study, we show that unloading by HS increased some elements of nuclear apoptotic signaling in the EDL because the increase in Bax protein abundance preceded any significant loss in muscle mass. Rather, in our hands, HS only had a modest decrease in EDL fiber area and there was no improvement in the recovery of fiber atrophy by Ca-HMB treatment in EDL after removing HS and reloading with the animal's body weight. The frequency of TUNEL-positive nuclei (myonuclei + satellite cells) increased in EDL after HS-mediated unloading, suggesting that other non-caspase-dependent apoptotic pathways may have been accelerated in EDL, although the increase in apoptosis signaling did not result in a loss of muscle mass during unloading. However, Ca-HMB has no net effect on reducing proapoptotic signaling or the frequency of TUNEL-positive nuclei in the EDL muscle for either HS or R conditions. Nevertheless, together, these data are supportive of the idea that apoptotic signaling precedes muscle loss in the EDL muscle mass and function during unloading. Future studies are needed to identify if Ca-HMB would have had a more important role in reducing apoptotic loss of myonuclei and/or satellite cells if apoptosis had been more severe and if the presumed threshold of lost nuclei had been reached so that muscle size could not be maintained in the EDL muscle.

### 4.4. Other HMB-Related Interventions

HS is a powerful atrophic stimulus for plantar flexors and particularly for the soleus muscle in rodents. Attempts to counter this loss of muscle mass and function in plantar flexor muscles by compounds related to HMB have included leucine and branch chain amino acids [150, 151] and taurine [152] which have been only partially effective as compared to HMB which has been more effective [43, 51]. For example, Ca-HMB appeared better than high levels of leucine in offsetting cancer cachexia and lowered protein degradation by reducing the activity of the ubiquitin-proteasome signaling [99]. However, this might be model dependent because another study has reported that Ca-HMB was unable to prevent the increased expression of E3 ubiquitin ligases Mafbx/Atrogin or improve tetanic peak force in the immobilized soleus muscles from dexamethasone-treated rats [153]. This suggests that Ca-HMB may be effective to attenuate the extent of atrophy in some models of muscle loss (plantaris, gastrocnemius unloading) but not all conditions that invoke muscle wasting.

## 5. Summary and Conclusion

In this study, we found that Ca-HMB was unable to change EDL mass or function or decrease apoptotic signaling in response to HS or R in aged rats. While multiple studies have shown that Ca-HMB was beneficial by suppressing pathways associated with muscle wasting and aging in the plantar flexor muscles (gastrocnemius, plantaris, and soleus muscles) of rodents [43, 51, 60, 62] and in the limb muscles of humans [53, 59, 80, 95, 103, 126–130, 141], other studies suggest that Ca-HMB could not reverse a decline in muscle function, mitochondria protein synthesis, or anabolic signaling in limb muscles with aging [136, 153, 154]. However, it is also possible that even though HMB might not improve muscle mass or function in aging, other benefits such as improved cognitive function which were not measured in the current study could be achieved [154]. We cannot exclude the possibility that if we had used a different model of disuse which caused greater muscle atrophy in the EDL (e.g., immobilizing the EDL in a dorsiflexed position [125]), we might have found a greater effectiveness for Ca-HMB to suppress EDL muscle atrophy in old rats. This assumes that EDL is not resistant to the effects of Ca-HMB. Alternatively, we cannot rule out the possibility that if we provided a longer duration of supplementation with Ca-HMB, or added a resistance exercise to the reloading phase after HS, or provided higher therapeutic doses of Ca-HMB throughout the study, we may have improved the anabolic environment of EDL muscles of old rats in this study. This direction is consistent with a report that a long duration of HMB treatment slowed the progression of sarcopenia in limb muscles of humans [155] and long-term treatment with HMB has been proposed as a therapeutic to offset sarcopenia in elderly humans [80, 156]. Future studies should be conducted to compare models that have different severities of muscle loss in aging to establish if there is a therapeutic dose and/or duration threshold that is required to maximize Ca-HMB's benefit to prevent muscle loss or to recover from muscle wasting that is caused by aging and disuse.

## Data Availability

The experimental data used to support the findings of this study are available from the corresponding author upon request.
